# Electronic Health Records and Quality of Care: An Observational Study Modeling Impact on Mortality, Readmissions, and Complications

**DOI:** 10.1097/MD.0000000000003332

**Published:** 2016-05-13

**Authors:** Swati Yanamadala, Doug Morrison, Catherine Curtin, Kathryn McDonald, Tina Hernandez-Boussard

**Affiliations:** From the Stanford University School of Medicine (SY); Stanford University School of Medicine (DM, CC, TH-B), Department of Surgery; Stanford University School of Medicine (KM), Center for Primary Care and Outcomes Research; and Stanford University School of Medicine (TH-B), Biomedical Informatics, Stanford, CA.

## Abstract

Electronic health records (EHRs) were implemented to improve quality of care and patient outcomes. This study assessed the relationship between EHR-adoption and patient outcomes.

We performed an observational study using State Inpatient Databases linked to American Hospital Association survey, 2011. Surgical and medical patients from 6 large, diverse states were included. We performed univariate analyses and developed hierarchical regression models relating level of EHR utilization and mortality, readmission rates, and complications. We evaluated the effect of EHR adoption on outcomes in a difference-in-differences analysis, 2008 to 2011.

Medical and surgical patients sought care at hospitals reporting no EHR (3.5%), partial EHR (55.2%), and full EHR systems (41.3%). In univariate analyses, patients at hospitals with full EHR had the lowest rates of inpatient mortality, readmissions, and Patient Safety Indicators followed by patients at hospitals with partial EHR and then patients at hospitals with no EHR (*P* < 0.05). However, these associations were not robust when accounting for other patient and hospital factors, and adoption of an EHR system was not associated with improved patient outcomes (*P* > 0.05).

These results indicate that patients receiving medical and surgical care at hospitals with no EHR system have similar outcomes compared to patients seeking care at hospitals with a full EHR system, after controlling for important confounders.

To date, we have not yet seen the promised benefits of EHR systems on patient outcomes in the inpatient setting. EHRs may play a smaller role than expected in patient outcomes and overall quality of care.

## INTRODUCTION

It is thought that health information technology, particularly electronic health records (EHR), will improve quality and efficiency of healthcare organizations, from small practices to large groups.^1^ Given these potential benefits, the federal government encouraged EHR adoption under the Health Information Technology for Economic and Clinical Health (HITECH) Act. In response, many hospitals are striving to adopt these systems and demonstrate meaningful use. In 2013, 59% of US hospitals had some type of EHR system.^2^ The federal incentive program defined 3 stages for timely adoption of EHR use: stage 1 is EHR adoption, stage 2 is EHR data exchange, and stage 3 is using EHRs to improve patient outcomes.^3^ However, despite the widespread adoption of EHR systems, only about 6% of hospitals met all criteria of stage 2 meaningful use.^2–4^ Thus, implementing and developing meaningful use for EHRs is still an ongoing process in the American healthcare system.

Electronic health records were originally built for billing purposes, not for research and quality improvement efforts.^5^ Accordingly, the impact of EHRs on quality healthcare delivery has focused on physician performance and billing precision.^6^ EHR studies often concentrate on process quality metrics, analyzing physician-level variability, and guideline compliance, rather than overall quality improvement or patient outcomes.^7–11^ Some have suggested that EHRs have the potential to decrease medical errors by providing improved access to necessary information, better communication and integration of care between different providers and visits, and more efficient documentation and monitoring.^12^ Many have used EHRs to decrease prescribing errors by providing real time clinical decision support.^13–16^ Other recent studies have begun to use EHRs to track and monitor adverse patient outcomes such as catheter-associated urinary tract infections, deep vein thrombosis, or pulmonary embolism, providing critical data to improve patient safety outcomes.^17,18^

Therefore, while several studies have looked at changes in quality attributed to electronic healthcare systems, overall improvements in patient outcomes associated with EHR implementation are still not yet well documented. In particular, the effect of the implementation of an EHR system on inpatient adverse events, inpatient mortality and 30-day all cause readmission for specific surgical, and medical conditions has yet to be explored. We thus sought to determine the association of hospital level-EHR systems with important patient outcomes.

The objective of this study was to determine whether hospitals with fully implemented EHR systems had better patient outcomes compared to hospitals with partial or no implemented EHR system after controlling for other important patient and hospital characteristics. Our study provides new information about the relationship between the implementation of an EHR system and the quality of healthcare delivered in the inpatient setting.

## METHODS

### Data Source

We utilized discharge data from the 2011 State Inpatient Databases (SID), Healthcare Cost and Utilization Project (HCUP), Agency for Healthcare Research and Quality from Arkansas, California, Florida, Massachusetts, Mississippi, and New York.^19^ SID is an all-capture state database that allows linkage of patients overtime and contains information on patient characteristics, primary and secondary diagnoses, and procedures received. The SID database was linked to the 2011 American Hospital Association (AHA) annual survey database, which contains information on EHR utilization in different hospitals along with other important hospital characteristics.^20^

Patient safety indicators (PSI) are based on ICD-9-CM codes and Medicare severity diagnosis-related groups (DRGs), with specific inclusion and exclusion criteria determined by the Agency for Healthcare Research and Quality (AHRQ).^21^ Using the PSI software (version 4.5),^22^ we identified these adverse events in our dataset. Each PSI includes a unique denominator, numerator, and set of risk adjustors.^23,24^

### Study Population

Both surgical and medical patients from several diagnostic categories were included in the study. Specifically, surgical patients undergoing pulmonary lobectomy, open abdominal aortic aneurysm repair, endovascular abdominal aortic aneurysm repair, or colectomy were included. Medical patients receiving care for acute myocardial infarction, congestive heart failure, or pneumonia were included. These categories were chosen based on their frequency, contribution to patient comorbidities, and prevalence in the medical literature.

### Outcome of Interest

Our main outcomes of interest were inpatient mortality, 30-day all cause readmission rates, PSIs, and length of stay.

### Statistical Analysis

We utilized univariate regression analysis to develop descriptive statistics. A hierarchical regression model relating level of EHR utilization and quality of care was developed. The independent variables were level of EHR utilization (no EHR, partial EHR, or full EHR), patient demographics, comorbidities, and medical or surgical group. The dependent variables were mortality, readmissions, and complications (measured by PSIs). Relative-risk difference in differences analyses (DiD) were used to determine the effect of implementing an EHR system on quality of care.^25^ The difference-in-differences analyses combined pre–post and treatment–control comparisons to eliminate some types of potential confounding. To do so, the hospitals lacking EHR systems in 2008 were selected and were then split into 3 groups: those that gained full EHRs by 2011 (treatment 1), those that gained only partial EHRs by 2011 (treatment 2), and those that still had no EHRs in 2011 (control). Direct comparison of these groups might be biased by confounders: there must be reasons why some of the hospitals adopted EHRs while others did not, and those reasons might also impact the outcomes of interest, both before and after adoption. Therefore, each hospital's event rates in 2008 are compared with the same hospital's rates in 2011, and the changes in those rates are then used to compare each treatment (i.e., EHR adoption level) with control (no EHR adoption). This analysis assumes that, in the absence of the intervention, the groups would have parallel trends and common external shocks. The use of a modified-Poisson model in these analyses enables estimation of risk ratios rather than odds ratios.^26^ All statistical analyses were performed using STATA version 13.0, except for the difference-in-differences analyses, which were performed using both STATA and SAS version 9.3.^27^

## RESULTS

Patient characteristics are described in Tables 1 and 2. A total of 137,162 surgical patients and 311,605 medical patients were included. Medical and surgical patients sought care at hospitals reporting no EHR (3.5%), partial EHR (55.2%), and full EHR (41.3%). Of the surgical patients, 2.7% were treated in a hospital with no EHR, 55.9% were treated in a hospital with partial EHR, and 41.4% were treated in a hospital with full EHR. Of the medical patients, 3.9% were treated in a hospital with no EHR, 55.0% of patients were treated in a hospital with partial EHR, and 41.2% were treated in hospitals with full EHR.

**TABLE 1:**
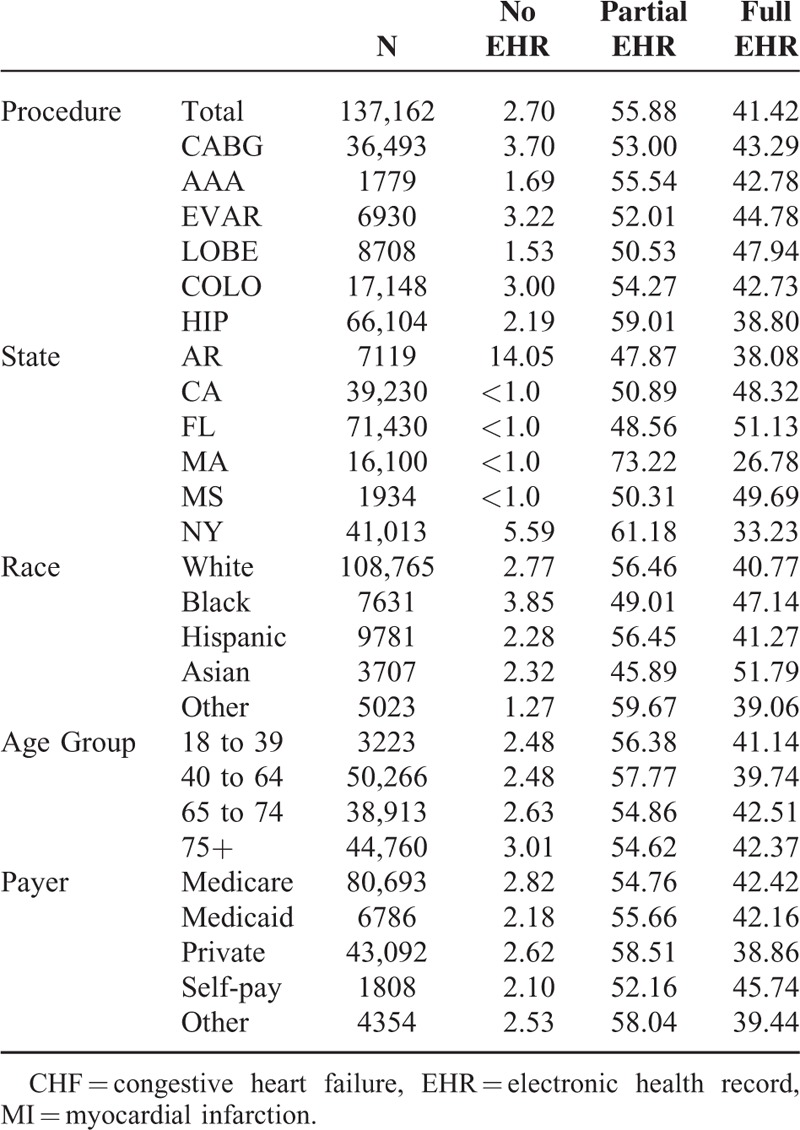
Surgical Patient Demographics by EHR Status, 2011

**TABLE 2:**
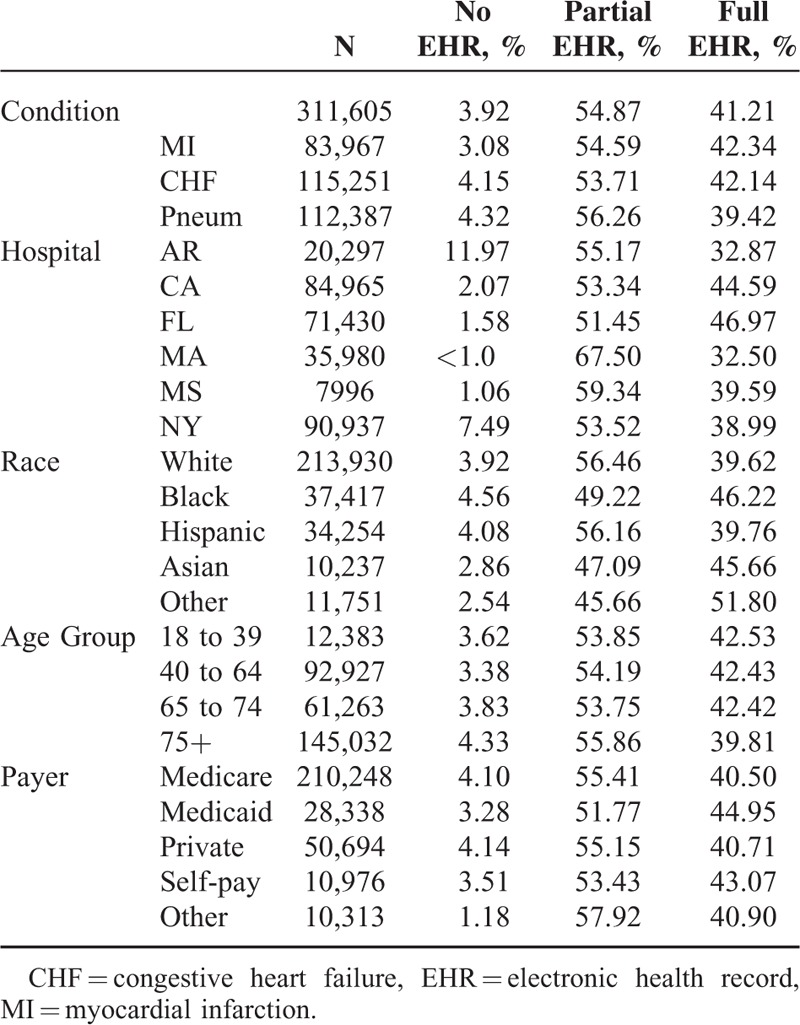
Medical Patient Demographics by EHR Status, 2011

In the cross-sectional analyses, surgical patients treated at hospitals with full EHR had higher mortality rates (1.6%) than patients treated at hospitals with partial EHR (1.4%) or at hospitals with no EHR (1.6%) (*P* = 0.0086). Surgical patients treated at hospitals with full EHR had higher readmission rates (11.9%) than patients treated at hospitals with partial EHR (11.2%) but lower readmission rates than patients treated at hospitals with no EHR (12.6%) (*P* = 0.0005). Surgical patients treated at hospitals with full EHR had higher rates of complications measured by PSIs (3.7%) than patients treated at hospitals with partial EHR (3.0%) or no EHR (3.2%) (*P* < 0.0001). Surgical patients treated at hospitals with full EHR had a shorter length of stay (LOS), measured in days (6.38) than patients treated at hospitals with partial EHR (6.85) or no EHR (7.89) (*P* < 0.0001) (Table 3).

**TABLE 3:**
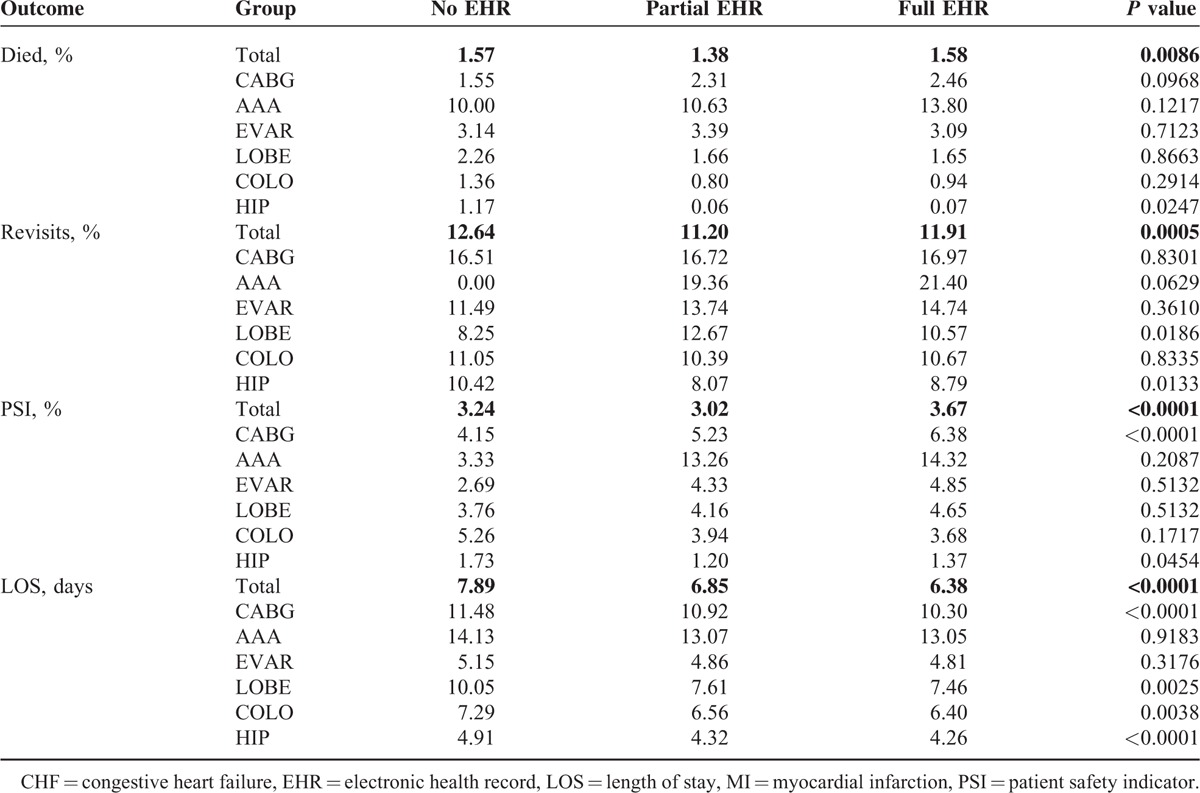
Cross-Sectional Univariate Analysis Surgical Patient Outcomes by EHR Status, 2011

Medical patients treated at hospitals with full EHR had a lower mortality rate (3.7%) than patients treated at hospitals with partial EHR (4.0%) or no EHR (4.4%) (*P* < 0.0001). Medical patients treated at hospitals with full EHR did not have a statistically significant different readmission rate (19.4%) compared to patients treated at hospitals with partial EHR (19.6%) or no EHR (20.3%) (*P* = 0.0548). Medical patients treated at hospitals with full EHR did not have a statistically significant difference in complications measured by PSIs (0.9%) compared to patients treated at hospitals with partial EHR (0.9%) or no EHR (0.8%) (*P* = 0.196). Patients treated at hospitals with full EHR had a shorter length of stay (5.02) than patients treated at hospitals with partial EHR (5.28) or no EHR (5.76) (*P* < 0.0001) (Table 4).

**TABLE 4:**
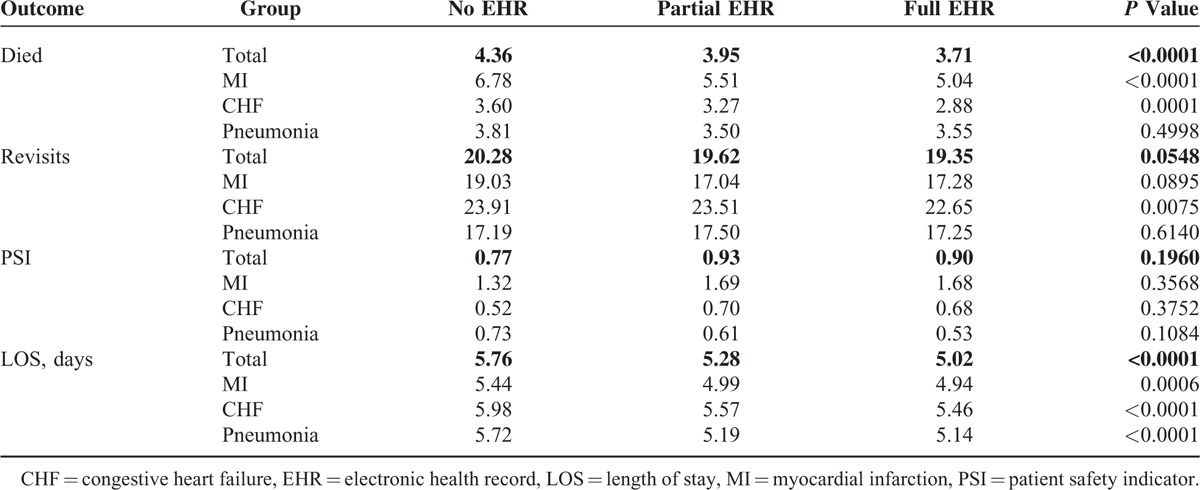
Cross-Sectional Univariate Analysis Medical Patient Outcomes by EHR Status, 2011

In the multiple regression analysis, there was no statistically significant difference in mortality rate among surgical patients treated at hospitals with full versus no EHR (odds ratio [OR] 1.24, *P* = 0.1442) or partial versus no EHR (OR 1.24, *P* = 0.1788) after controlling for important patient and hospital characteristics. There was no statistically significant difference in readmission rates among surgical patients treated at hospitals with full versus no EHR (OR 1.04, *P* = 0.5605) or partial versus no EHR (OR 1.04, *P* = 0.5158). There was a significant difference between rates of any complication measured by PSIs among surgical patients treated at hospitals with full versus no EHR (OR 1.22, *P* = 0.0452) but not between patients treated at hospitals with partial versus no EHR (OR 1.12, *P* = 0.2679).

There was no statistically significant difference between mortality rates among medical patients treated at hospitals with full EHR versus no EHR (OR 0.97, *P* = 0.4609) or partial versus no EHR (OR 1.01, *P* = 0.9375). There was no statistically significant difference between readmission rates among medical patients treated at hospitals with full versus no EHR (OR 0.97, *P* = 0.2834) or partial versus no EHR (OR 1.00, *P* = 0.8697). There was no statistically significant difference in complications measured by PSIs among medical patients treated at hospitals with full versus no EHR (OR 1.06, *P* = 0.6157) or partial versus no EHR (OR 1.13, *P* = 0.2516) (Table 5).

**TABLE 5:**
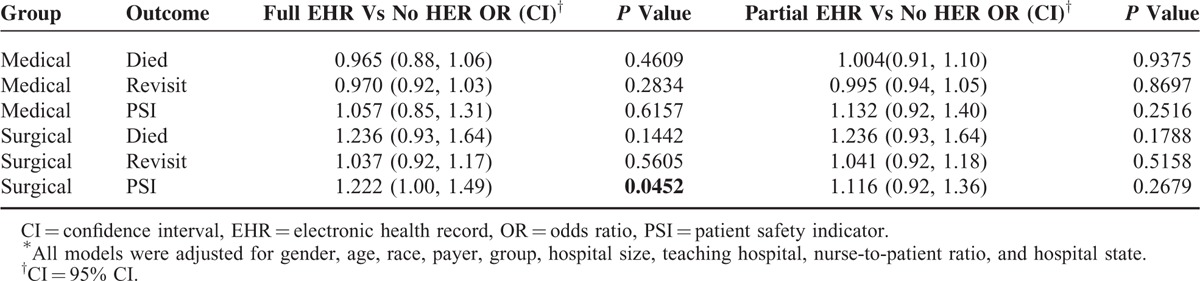
Association Between Patient Outcomes and EHR Implementation Status^∗^

The difference-in-differences analysis allowed us to estimate the effect of implementing an EHR system on patient outcomes, assuming that the parallel trends and common shocks assumptions are correct. These analyses found statistically significant evidence of an effect in only three cases (Table 6).

**TABLE 6:**
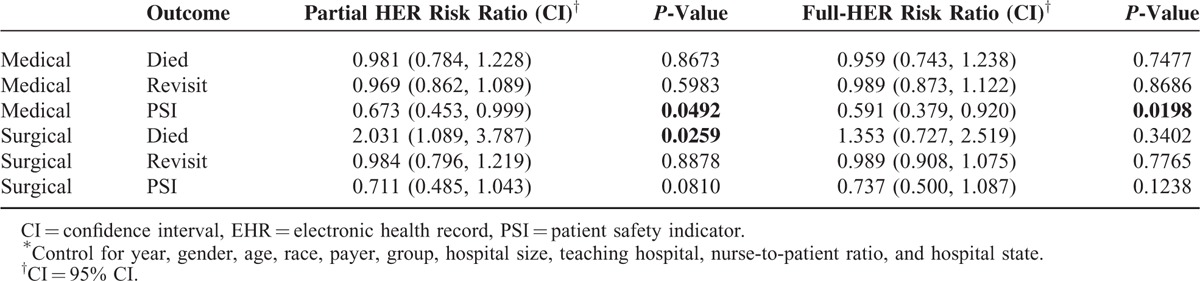
The Relative Risk^∗^ of EHR-Adoption on Patient Outcomes, 2008 and 2011

There was evidence of reduced risk of PSIs for medical patients in hospitals that had partially implemented EHRs by 2011 compared to those that still lacked EHRs in 2011 (risk ratio 0.673, 95% confidence intervals [CI] [0.45, 1.00], *P*-value 0.0492). There was also evidence of reduced risk of PSIs for medical patients in hospitals with fully implemented EHRs by 2011 compared with hospitals with no EHRs in 2011 (risk ratio 0.591, CI [0.38, 0.92], *P*-value 0.0198. Finally there was evidence of reduce risk of inpatient mortality for surgical patients in hospitals with partially implemented EHRs compared with no EHRs (risk ratio 2.031, CI [1.09, 3.79], *P*-value 0.0259) (Table 6).

## DISCUSSION

This study tested the association between level of EHR implementation in inpatient settings and patient outcomes across 6 large, diverse states for both medical and surgical care. These results provided a preliminary glimpse at EHR meaningful use. Cross-sectional analysis found significant differences in rates of mortality, readmission, and complications between patients at hospitals with full EHR or partial EHR compared to hospitals with no EHR. However, these differences did not hold when adjusted for patient and hospital factors. Furthermore, the effect of EHR adoption was not associated with improved patient outcomes (specifically inpatient mortality, readmissions, and complications). Although EHR systems are thought to improve quality of care, this study suggests that in their current form, EHRs have not begun to reach meaningful use targets and may have a smaller impact than expected on patient outcomes.

This study builds on multiple studies highlighting the limitations of EHR systems on improving quality of care. Although EHRs have been extremely helpful for billing and physician compliance measurements, direct improvement of important patient outcomes have yet to be seen. A possible reason for this is that EHRs thus far have largely served as a recording mechanism after a patient care intervention rather than as an effective checking mechanism during the actual execution phase of patient care interventions. It has also been shown that while basic EHRs are associated with gains in quality measures, less benefit is associated with implementing advanced EHRs, suggesting that initial adoption of EHRs may actually be counterproductive by adding additional complexity into clinical settings.^28^ Additionally, such improvements have yet to be translated to improvements in mortality.^29^ Lack of improvement in other patient outcome measures has also been demonstrated. For example, 1 study demonstrated that although EHRs were associated with better rates of cholesterol testing, this did not translate to improvements in patients’ actual cholesterol levels.^9^ In another study of ambulatory diabetes care in clinics with and without EHRs, patients at EHR enabled clinics actually did worse in rates of meeting 2 year hemoglobin A1c, cholesterol, and blood pressure goals.^30^ Furthermore, data suggest that EHR implementation may actually increase the amount of time spent by patients during clinic visits.^31^ These studies suggest that EHRs, with their increased documentation requirements, can have unintended consequences, including clinic inefficacy. All of these studies highlight the complexity of quality of care. Accurate documentation and billing is easily obtainable with EHRs but improving recognition of clinical problems and changing provider practice is much more challenging.

Our study does have some limitations. This study relied on administrative data derived from billing claims data, which has limited information on patients’ treatment courses that can affect outcomes. Additionally, this study uses hospital survey data to identify the level or EHR adoption, which is prone to reporting errors. Furthermore, changes in quality of care after the implementation of EHRs may be attributable in part to non-EHR factors, which cannot be fully accounted for in our analysis.

This population-based study builds on existing literature to demonstrate that EHRs are not yet associated with gains in measures of inpatient mortality, readmissions, and PSIs. Results here suggest that differences in outcomes at hospitals with different levels of EHR utilization may be attributable to other patient and hospital factors rather than EHR utilization itself. As federal incentives encourage EHR adoption and hospitals strive for meaningful use, it will be important to further characterize the benefits received from EHRs.
